# Cryotherapy Improves Limb Use But Delays Normothermia Early After Stifle Joint Surgery in Dogs

**DOI:** 10.3389/fvets.2020.00381

**Published:** 2020-07-03

**Authors:** Stephanie D. Szabo, David Levine, Denis J. Marcellin-Little, Brian K. Sidaway, Erik Hofmeister, Erica Urtuzuastegui

**Affiliations:** ^1^College of Veterinary Medicine, Midwestern University, Glendale, AZ, United States; ^2^Department of Physical Therapy, University of Tennessee at Chattanooga, Chattanooga, TN, United States; ^3^Department of Veterinary Surgical and Radiological Sciences, School of Veterinary Medicine, University of California, Davis, Davis, CA, United States; ^4^Incise Veterinary Surgery, Peoria, AZ, United States; ^5^College of Veterinary Medicine, Auburn University, Auburn, AL, United States

**Keywords:** dog, cryotherapy, stifle joint, pressure pain threshold (PPT), weight bearing, canine rehabilitation, TPLO

## Abstract

**Objective:** To evaluate the short-term efficacy and safety of cold compression therapy (CCT) relative to a soft padded bandage (SPB) in dogs undergoing surgery to manage cranial cruciate ligament injury.

**Methods:**Dogs were randomized into groups that received CCT or SPB after surgery. Weight bearing was measured using a weight distribution platform before and the day after surgery. Stifle joint flexion and extension were measured using a goniometer before and the day after surgery. Rectal temperatures were measured every 15 min for 2 h after surgery and the morning after surgery. Mechanical nociceptive thresholds (MNT) were measured using an algometer the day after surgery. Findings in both groups were compared using a mixed model ANOVA.

**Results:**20 dogs were enrolled: 10 in the CCT and 10 in the SPB group. Dogs undergoing CCT had more stifle joint flexion (*P* = 0.008) and weight bearing (*P* < 0.001) after surgery than dogs with SPB. MNT after surgery correlated statistically with stifle joint flexion after surgery (*r* = −0.315, *P* = 0.014), extension after surgery (*r* = 0.310, *P* = 0.016), and weight bearing after surgery (*r* = 0.314, *P* = 0.003). Return to normothermia was delayed in the CCT group, with temperatures ~0.5°C (1.0°F) lower 105 (*P* = 0.018) and 120 min (*P* = 0.013) after surgery.

**Conclusion:**Relative to bandaging, CCT had a positive short-term impact on stifle flexion and weight bearing. CCT delayed warming after surgery but dogs were only mildly hypothermic [0.5°C [1.0°F]].

## Introduction

Cryotherapy is commonly used postoperatively with the intent to slow tissue metabolism, (1, 2) to decrease edema and pain, (1, 3–5) to improve function, (1, 4, 6–10) and to decrease the need for medications (6, 7, 9). Clinically, cryotherapy is routinely used, with or without compression, after tibial plateau leveling osteotomy (TPLO) surgery. Cryotherapy applied over the stifle joint reduces intra-articular temperature and cryotherapy over epaxial muscles decreases muscle temperature (11, 12). Optimal treatment time appears to range from 10 to 20 min. In one study evaluating dogs after stifle joint surgery, cryotherapy was more effective than a soft padded bandage (SPB) in minimizing edema (5). In a blinded, randomized controlled trial involving dogs undergoing TPLO surgery, 4 sessions cold compression therapy (CCT) over the first 24 h decreased pain, increased range of motion, and improved lameness (4). Similarly, in a pilot study of dogs undergoing TPLO surgery, a potential increase in weight bearing was present in dogs after cryotherapy or after CCT, compared to a SPB (13).

In humans, anesthesia reduces core temperature by 0.5–1.5°C in the first 30 min after induction (14–16). Similarly, the majority of dogs are hypothermic after anesthesia because of impaired thermoregulatory control (17). It is unclear, however, whether cryotherapy immediately after surgery delays the recovery of core temperature in dogs. The purpose of the study presented here was to compare the post-operative effects of CCT or a SPB on stifle flexion and extension, weight bearing, mechanical nociceptive thresholds (MNT), and changes in core temperature after stifle joint surgery.

## Materials and Methods

Client-owned dogs with cranial cruciate ligament injury scheduled for TPLO or tibial tuberosity advancement (TTA) surgery were included in the study. The study protocol was approved by the institutional animal care and use committee (MWU #2884). Client signed an informed consent. Dogs were excluded if they weighed <22 kg or more than 45 kg, if they had bilateral cranial cruciate disease, severe orthopedic disease in another joint, or had previously undergone surgery of the operated joint. Demographic information (date of birth, sex, breed, stifle affected) and a complete physical examination were performed by the same investigator (S.D.S.) at screening. Study patients were then randomly assigned by coin toss to one of two groups: CCT using a cold compression device or a SPB.

Weight distribution was measured five times before surgery on the day of surgery with measurements acquired a few seconds apart using a weight distribution platform^1^. Flexion and extension of the stifle joint was measured by one investigator (E.U.) in triplicate with measurements collected 10 s apart using a plastic goniometer and a standardized method (18). Dexmedetomidine^2^ (5 μg/kg) and hydromorphone^3^ (0.1 mg/kg) were administered intramuscularly for premedication. Anesthesia was induced with propofol^4^ (4 mg/kg given intravenously to effect) and maintained with isoflurane^5^ in 100% oxygen. Regional anesthesia was provided with lumbosacral epidural morphine^3^ (0.1 mg/kg) and bupivacaine^6^ (0.5 mg/kg of a 0.5% solution). A TPLO or TTA was performed on the affected stifle, as previously described (17, 19). The surgical procedure was selected based on surgeon's preference.

Carprofen^4^ (4.4 mg/kg) or meloxicam^7^ (0.1 mg/kg) was administered subcutaneously after surgery. Depending on opioid availability, hydromorphone (0.05 mg/kg) was administered subcutaneously every 5 h or an intravenous continuous rate infusion of fentanyl^8^ (2–3 μg/kg/h) was administered after surgery. Forced air warming^9^ was used throughout recovery. Rectal temperatures were recorded every 5 min during surgery (data not reported). For dogs in the SPB group, a soft padded bandage was placed around the operated leg after surgery. The primary contact layer was a non-adhesive pad over the surgical incision^10^. Rolled cotton^11^ and gauze^12^ formed the secondary layer. Self-adhesive tape^13^ formed the outer layer. The bandage was removed at 6:00 AM the morning after surgery. For dogs in the CCT group, a cold compression sleeve was placed around the operated leg after surgery. The cold compression unit^14^ delivered 30 min of medium cold compression (5–50 mm Hg, 2–3 min of inflation, 1 min of deflation) every 2 h at 3.3°C (38.0°F). CCT was discontinued at 6:00 AM the morning after surgery. Rectal temperatures were measured with a calibrated thermometer and recorded every 15 min after surgery for 2 h or until the patient's body temperature reached 37.2°C (99.0°F). Rectal temperatures were recorded at 7:00 AM the day after surgery.

Twenty-four hours after surgery, weight distribution was measured five times using the weight distribution platform. Flexion and extension of the stifle joint were measured in triplicate. A pressure algometer^15^ was used by one investigator (E.U.) to measure MNT. Three measurements were recorded 10 s apart while the dog was in lateral recumbency with the operated leg up. A positive response was a limb withdrawal or an unequivocal pain response.

The initial motivation for the study was to evaluate whether CCT would delay normothermia after surgery. Historical temperature data were used to perform a power analysis^16^ to detect a 0.35°C difference between groups at a power of 0.80 and an alpha of 0.05. The power analysis yielded a sample size of 10 dogs per group. Postoperative rectal temperature over time was assessed using a mixed model analysis of variance that accounted for repeated measures within dogs^17^. Weight bearing, flexion, and extension were analyzed using mixed model analysis of variance that accounted for repeated measures within dogs. The effects of treatment (bandage/CCT) and the effect of surgery (before/after surgery) and all possible interactions between those factors were included in the model. Mechanical nociceptive threshold measurements were analyzed with an ANOVA, accounting for repeated measures within dogs. Cohen's *d* were calculated to assess normalized effect sizes between treatment groups: *d* values < 0.2 were deemed negligible, *d* > 0.2 and ≤ 0.5 were *small, d* > 0.5 and ≤ 0.8 were *medium*, and *d* > 0.8 were *large* (20). For all measures, normality of the responses was confirmed by assessing the distribution of residuals using Shapiro-Wilks tests. Pairwise comparisons were made between treatments. Relationships between weight bearing, stifle flexion and extension, and algometry were evaluated using Pearson's correlations. For all tests, differences in least squared means with *P* < 0.05 were considered statistically significant.

## Results

Twenty dogs completed the study: 10 in the CCT group and 10 in the SPB group. Complications were not identified during the study period in any dog. Stifle joint flexion, extension, the weight placed on the operated limb and MNT are reported in Table 1. After surgery, dogs undergoing CCT had more stifle joint flexion (*P* = 0.008) and were bearing more weight (*P* < 0.001) on their operated limb than dogs with SPB. These differences represented a *medium* positive effect on stifle joint flexion and weight bearing. Compared to SPB, CCT also had a *small* positive effect on stifle joint extension and on MNT (20). After surgery, MNT correlated statistically with stifle joint flexion (negative correlation, *r* = −0.315, *P* = 0.014), extension (positive correlation, *r* = 0.310, *P* = 0.016), and weight bearing (positive correlation, *r* = 0.314, *P* = 0.003) but MNT after surgery did not correlate statistically with preoperative stifle joint flexion (*r* = 0.167, *P* = 0.201) or extension (*r* = −0.242, *P* = 0.063).

**Table 1:** Least squared means (± SEM) joint motion and limb use before and after stifle joint surgery in 20 dogs undergoing stifle joint surgery treated with a soft padded bandage (*n* = 10) or cold compression therapy (*n* = 10).

**Variable**	**Soft padded bandage**		**Cold Compression Therapy**	
	**Before surgery**	**After surgery**	**Before surgery**	**After surgery**	Cohen's d^*^
Stifle flexion (degrees)	52.5 n^a^ ± 1.9	61.1^b^ ± 1.9	49.6 ± 1.9	53.6 ± 1.9^†^	0.63
		(*P =* 0.001)		(*P =* 0.143)	
Stifle extension (degrees)	157.5^a^ ± 2.3	146.6^b^ ± 2.3	160.9^c^ ± 2.3	150.9^d^ ± 2.3	0.34
		(*P =* 0.001)		(*P =* 0.002)	
Weight on operated limb (%BW)	10.4 ± 0.9%	8.2 ± 0.9%	13.9 ± 0.9%	12.7% ± 0.9%^†^	0.65
		(*P =* 0.084)		(*P =* 0.358)	
Pressure pain threshold (kPa)		166 ± 7.12		179 ± 8.01	0.26

* *effect size: difference in treatment effect between soft padded bandage and cold compression therapy after surgery*.

† *means after surgery differ between the soft padded bandage and cold compression therapy groups*.

a-d *Within rows and within each treatment group, means with different superscript letters differ statistically before and after surgery (P-values for comparisons are included)*.

*%BW, Percent of body weight*.

**Figure 1:**
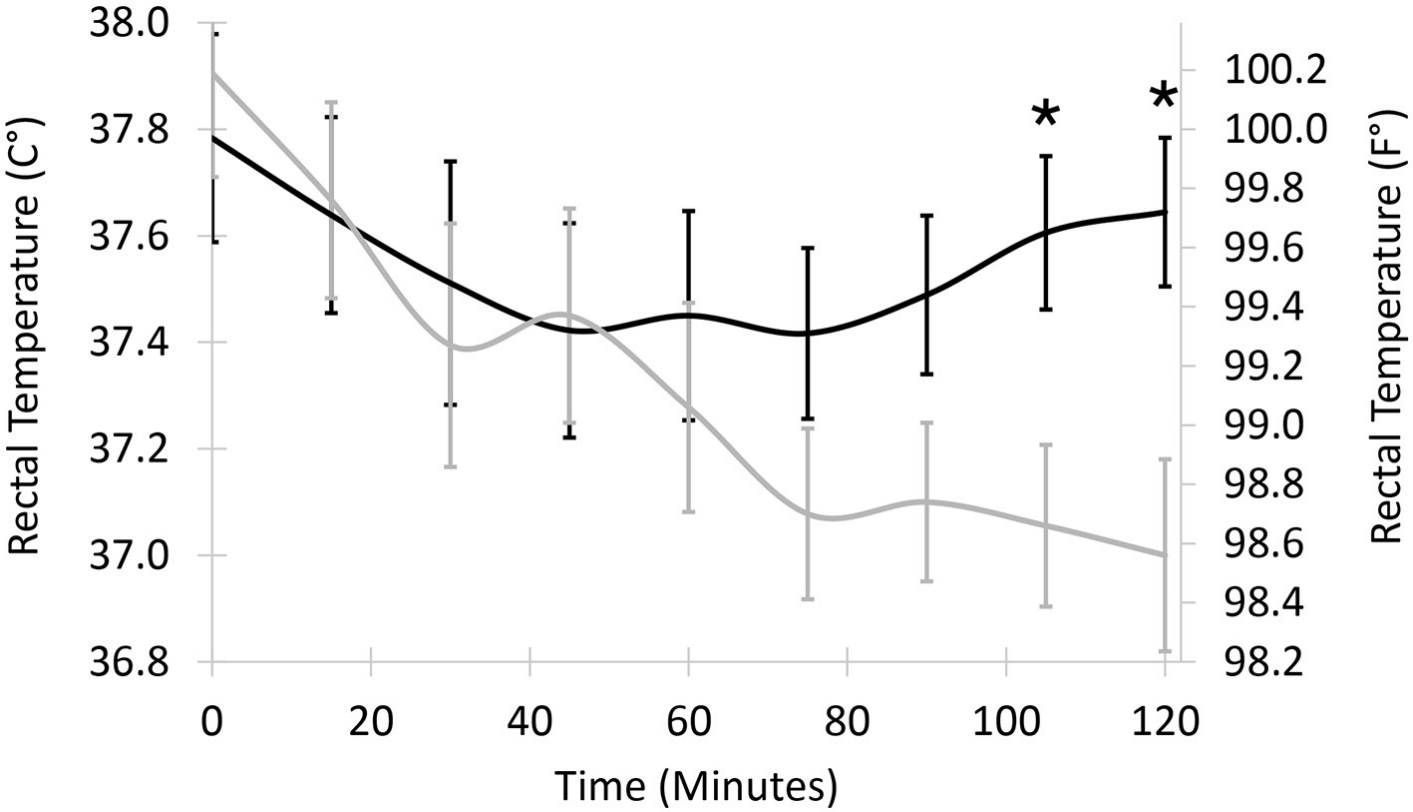
Least squared mean core temperature in 20 dogs undergoing stifle joint surgery treated with a soft padded bandage (*n* = 10, black line) or cold compression therapy (*n* = 10, gray line). Vertical bars represent standard errors to the means. Temperatures differ statistically between least squared means 105 and 120 min after surgery (asterisks, *P* = 0.018 and 0.013, respectively).

Mean core temperatures ranged from 37.8°C (100.0°F) at the end of surgery to 37.4°C (99.4°F) 75 min after surgery for dogs in the SPB group and from 37.9°C (100.2°F) at the end of surgery to 37.0°C (98.6°F) 120 min after surgery for dogs in the CCT group. Mean core temperatures did not differ statistically at early time points but were lower for CCT than SPB 105 min after the end of surgery (37.1 vs. 37.6°, *P* = 0.018, Figure 1) and 120 min after the end of surgery (37.0 vs. 37.6°, *P* = 0.013). The morning after surgery, mean rectal temperatures in the CCT group (100.5°C) did not differ from the SPB group (100.8°C, *P* = 0.518).

## Discussion

As anticipated, surgery led to a decrease in weight bearing in operated limbs the day after surgery. However, dogs undergoing CCT were bearing more weight on their operated limb and had more flexion of their operated joint than dogs that were bandaged the day after surgery. CCT had a medium effect size on weight bearing. This is in agreement with two previous reports that documented an improvement in weight bearing in dogs receiving CCT after stifle joint surgery (4, 21). Increased weight bearing of the operated limb likely was the result of a decrease in perceived pain and a potential decrease in periarticular edema and joint effusion. The increased post-operative weight bearing on the operated limb in patients undergoing CCT compared to patients with SPB suggests that the recovery of dogs undergoing CCT could be accelerated and could decrease the severity of tissue changes associated with limb disuse during recovery from surgery.

While surgery led to a loss of stifle joint flexion, dogs undergoing CCT had more flexion than dogs that were bandaged, most likely because of a decrease in periarticular swelling and possibly a decrease in joint effusion and in pain. This is in agreement with a previous study in the dog stifle where cold compression was more effective than bandaging to control swelling (5). Cryotherapy has been shown to improve flexion in humans undergoing knee surgery, possibly because of enhanced muscle control in patients undergoing cryotherapy (22). The increased joint motion in dogs in patients undergoing CCT compared to patients with SPB could protect dogs against periarticular fibrosis resulting and is likely to improved patient comfort and limb use.

In the current study, pain was evaluated using MNT. While mean MNT did not differ statistically among treatment groups, the combination of a numerically higher MNT in the CCT group (179 kPa) compared to the bandage group (166 kPa) combined with a small positive effect suggests that CCT may have had a small impact on MNT. The statistically significant correlations between MNT, stifle joint flexion, stifle joint extension, and weight bearing on the operated limb confirm the association of higher nociceptive thresholds with increased joint motion and increased limb use. CCT may have led to decreased edema, leading to increased joint motion. The combination of increased nociceptive thresholds and increased joint motion may have led to increased limb use. These findings are in agreement with findings in humans with knee OA. In one study, continuous compression increased MNT (23). Cryotherapy also increases pain thresholds, in part because it decreases nociceptive conduction velocity (24). MNT is form of quantitative sensory testing, alongside mechanical (von Frey filament, pinprick, vibration) and thermal (hot, cold) testing options. The use of quantitative sensory testing to evaluate hyperalgesia and allodynia is increasing in experimental animals, in client-owned animals, and in humans with post-operative and chronic osteoarthritic pain (25–27). In one study of people with knee osteoarthritis, MNT measurements were superior to visual analog scores to evaluate knee pain (28). Pressure pain thresholds are decreased in humans with knee osteoarthritis and appear associated with symptom severity (29). In one study in dogs with hip osteoarthritis, MNT was decreased at the time of surgery and returned to normal 3 months after successful joint replacement (26). It is not clear from this short-term study whether post-operative differences in MNT would influence limb use during recovery beyond the first day and in the long term. In humans, post-operative pain thresholds are associated with recovery: several forms of altered thresholds immediately after surgery predict long-term chronic pain (30). Also, MNT remain low in individuals who report persistent pain after surgery (31). Further evaluation is warranted to confirm that MNT are positively impacted by CCT and to evaluate the duration of efficacy and potential long-term impact of CCT.

The dogs in the study were mildly hypothermic at the end of surgery, most likely because active measures were used to prevent severe hypothermia (32). CCT had a measurable influence on core temperature after surgery. For dogs with bandages, the low temperature trough appeared to occur between 45 and 75 min after surgery. By comparison, the temperature in dogs with CCT decreased during the 2-h post-operative period. Because temperature monitoring stopped 2 h after surgery, when and what the low temperature trough was for dogs in the CCT group and how rapidly temperatures returned to normal values is not known. However, temperature differences between groups were small, ~0.5°C (1°F), and the lowest body temperatures were only mildly hypothermic (lower end of the 95% confidence intervals for post-operative temperatures ranging from 36.6 to 37.5°C, with mild hypothermia defined as temperature ranging from 36.5 to 38.5°C) (17). With mild hypothermia, pharmacodynamic changes for pain medications would likely be minor (33). Clinical signs of hypothermia in dogs receiving CCT were not detected during recovery. The investigators had the clinical impression that the cold compression was well-tolerated by patients and did not disrupt rest after surgery. Temperature differences between the SPB and CCT group were no longer present the following morning. In dogs, focal skin surface cooling has limited systemic impact because of the limited surface area for heat exchange (34). In one study in dogs, focal ice compresses applied to the knee for 5, 15, or 30 min had no systemic impact, while immersion of the joint in an ice bath for 15 min led to a decrease in rectal temperature of 1.6 ± 0.3°C (11). The delay in recovery of systemic temperature in the current study may be due to the increased efficacy of CCT compared to ice compresses. Efficacy was likely enhanced by the presence of the cold reservoir of the recirculating unit and by compression (35).

This study had limitations. Clinical factors such as chronicity of cranial cruciate ligament damage, severity of articular and periarticular fibrosis, and severity of stifle joint osteoarthritis were not recorded in the current study. A larger prospective study including measures of chronicity, articular fibrosis, osteoarthritis, and including longer-term outcome measures would provide more comprehensive information on the benefits of CCT for patients undergoing stifle joint surgery. Weight bearing was evaluated statically, with the dogs standing on a weight distribution platform. Most objective evaluations of gait involve a force plate or a pressure sensitive walkway rather than a weight distribution platform. It is unclear how differences in static weight bearing would translate to differences in dynamic weight bearing at a walk or trot on a force plate or pressure sensitive walkway. The effects of the surgical procedure (TPLO or TTA) were not included in the analyses because they were assumed to have no influence on the relative efficacy of CCT and bandaging. The relative efficacy of CCT and bandaging could possibly vary based on specific surgical procedures.

In conclusion, CCT had a positive short-term impact on stifle flexion and early weight bearing relative to bandaging. These findings are in agreement with previous reports (4, 21). Their long-term impact should be evaluated further. CCT appeared to delay warming after surgery. The delay was minor: dogs were only mildly hypothermic.

## Data Availability Statement

The datasets presented in this study can be found in online repositories. The names of the repository/repositories and accession number(s) can be found in the article/Supplementary Material.

## Ethics Statement

The animal study was reviewed and approved by Midwestern University College of Veterinary Medicine IACUC committee. Written informed consent was obtained from the owners for the participation of their animals in this study.

## Author Contributions

We certify that all authors meet the qualifications for authorship as listed below: DL, SS, and EH: substantial contributions to the conception or design of the work. EU and SS: acquisition. DM-L: analysis. DM-L, EH, and EV: interpretation of data for the work. DM-L, EH, and DL: drafting the work or revising it critically for important intellectual content. All authors: agreement to be accountable for all aspects of the work in ensuring that questions related to the accuracy or integrity of any part of the work are appropriately investigated and resolved. All authors contributed to the article and approved the submitted version.

## Conflict of Interest

The authors declare that the research was conducted in the absence of any commercial or financial relationships that could be construed as a potential conflict of interest.

## Footnotes

^1^Stance Analyzer, Companion Animal Health, Newark, DE

^2^Orion Corporation, Espoo, Finland

^3^Hikma, Eatontown, NJ

^4^Zoetis, Parsippany, NJ

^5^VetOne, Meridian, ID

^6^Hospira, Lake Forest, IL

^7^Boehringer Ingelheim, Fremont, CA

^8^Akorn, Lake Forest, IL

^9^Baja Breeze Warm Air Blower, Animal Hospital Supply, Flowery Branch, GA

^10^Telfa pad, Covidien, Medtronic, Minneapolis, MN

^11^Curity rolled cotton, Covidien, Medtronic, Minneapolis, MN

^12^Rolled gauze, Medline, Northfield, IL

^13^VetRap, 3M, Maplewood, MN

^14^Veterinary GRPro 2.1 Game Ready Control Unit, CoolSystems, Concord, CA

^15^Wagner Force Ten, Wagner Instruments, Greenwich, CT

^16^Power and Sample Size, http://powerandsamplesize.com/Calculators/Compare-2-Means/2-Sample-Equality, Hylown Consulting, Atlanta, GA, accessed May 18, 2020

^17^Proc Mixed, SAS, SAS Institute, Cary NC
